# Assessment of Pre-operative Measurements of Tumor Size by MRI Methods as Survival Predictors in Wild Type IDH Glioblastoma

**DOI:** 10.3389/fonc.2020.01662

**Published:** 2020-09-02

**Authors:** Alexis Palpan Flores, Catalina Vivancos Sanchez, José M. Roda, Sebastian Cerdán, Andres Javier Barrios, Cristina Utrilla, Aranzazu Royo, Maria Luisa Gandía González

**Affiliations:** ^1^Department of Neurosurgery, University Hospital La Paz, Madrid, Spain; ^2^Institute of Biomedical Research “Alberto Sols” CSIC/UAM, Madrid, Spain; ^3^Department of Neuroradiology, University Hospital La Paz, Madrid, Spain

**Keywords:** glioblastoma, overall survival, progression free survival, IDH mutation, tumor volumetry, linear method, semi-automatic segmentation method, ellipsoid method

## Abstract

**Objective:** We evaluate the performance of three MRI methods to determine non-invasively tumor size, as overall survival (OS) and Progression Free Survival (PFS) predictors, in a cohort of wild type, IDH negative, glioblastoma patients. Investigated protocols included bidimensional (2D) diameter measurements, and three-dimensional (3D) estimations by the ellipsoid or semi-automatic segmentation methods.

**Methods:** We investigated OS in a cohort of 44 patients diagnosed with wild type IDH glioblastoma (58.2 ± 11.4 years, 1.9/1 male/female) treated with neurosurgical resection followed by adjuvant chemo and radiotherapy. Pre-operative MRI images were evaluated to determine tumor mass area and volume, gadolinium enhancement volume, necrosis volume, and FLAIR-T_2_ hyper-intensity area and volume. We implemented then multivariate Cox statistical analysis to select optimal predictors for OS and PFS.

**Results:** Median OS was 16 months (1–42 months), ranging from 9 ± 2.4 months in patients over 65 years, to 18 ± 1.6 months in younger ones. Patients with tumors carrying O^6^-methylguanin-DNA-methyltransferase (MGMT) methylation survived 30 ± 5.2 vs. 13 ± 2.5 months in non-methylated. Our study evidenced high and positive correlations among the results of the three methods to determine tumor size. FLAIR-T_2_ hyper-intensity areas (2D) and volumes (3D) were also similar as determined by the three methods. Cox proportional hazards analysis with the 2D and 3D methods indicated that OS was associated to age ≥ 65 years (HR 2.70, 2.94, and 3.16), MGMT methylation (HR 2.98, 3.07, and 2.90), and FLAIR-T_2_ ≥ 2,000 mm^2^ or ≥60 cm^3^ (HR 4.16, 3.93, and 3.72), respectively. Other variables including necrosis, tumor mass, necrosis/tumor ratio, and FLAIR/tumor ratio were not significantly correlated with OS.

**Conclusion:** Our results reveal a high correlation among measurements of tumor size performed with the three methods. Pre-operative FLAIR-T_2_ hyperintensity area and volumes provided, independently of the measurement method, the optimal neuroimaging features predicting OS in primary glioblastoma patients, followed by age ≥ 65 years and MGMT methylation.

## Introduction

Glioblastomas are the most aggressive and frequent primary tumors of the central nervous system in adult populations, with an approximate incidence of 3–5/100,000 people (1, 2). Overall Survival (OS) predictions around 12–18 months are normally expected, remaining poor despite surgical, and adjuvant chemo- and radio-therapy treatments (3, 4). Glioblastomas are currently classified in primary or secondary subtypes, reflecting either a “*de novo*” origin without evidence of any preceding lesion or, a “progression from a lower grade astrocytoma,” respectively (5). Both subtypes have very different OS profiles, with longer survival estimates for the secondary. The molecular signature discriminating between primary (IDH wild type), or secondary subtypes, is the absence or presence, of the isocitrate dehydrogenase (IDH) mutation (5–7), respectively. Methylation of the O6 methylguanine DNA Methyltransferase (MGMT) promoter represents the next molecular feature in prognostic relevance, predicting a better response to alkylating agents like temozolamide (TMZ), during adjuvant chemotherapy (8, 9).

Both, clinical and neuroimaging variables, have been repeatedly recommended as prognostic factors (10, 11). More specifically, non-invasive measurements of the tumor size have played a controversial role in OS and Progression Free Survival (PFS) predictions of glioblastoma patients (12–19). Briefly, Magnetic Resonance Imaging (MRI) measurements of two-dimensional (2D) diameters of the tumor through the imaging plane were recommended initially by Macdonald et al. (20), and later incorporated by the Response Assessment Neuro-Oncology (RANO) working group (21), as criteria of response to therapy. However, the efficacy of the 2D protocol remains currently under debate, particularly, in morphologically irregular tumors (22–24). Almost in parallel, the 3D ellipsoid method, calculating the tumor volume (25, 26) and using three orthogonal diameters, was implemented for different tumors, including glioblastoma. However, the irregular shape of most tumors continued to limit accurate volume determinations using this approach (24, 27). More recently, advanced 3D image-processing software packages using semi-automatic segmentation algorithms have become available, providing more accurate estimations of tumor volumes, with the ability to measure irregular tumor shapes and their compartments (22, 28). Nevertheless, a main question arises on whether this increased accuracy in the volume measurement results in any benefit in terms of prognosis, as compared to the other two, simpler to implement, methods (23).

Notably, OS and PFS in glioblastomas have been previously associated to various compartmental tumor volumes including: the volume of necrosis (12–14), the volume of contrast-enhancing tumor (11), the volume of FLAIR-T_2_ hyper-intensity (13–18), or the tumor/necrosis volume ratio (13, 19). Unfortunately, results and conclusions from many of these studies remain contradictory. Discrepancies may originate, at least partially, from the diversity of volumetric techniques implemented and, in some cases, from incomplete morphological or molecular characterizations of the tumors investigated. Together, these limitations result in a plethora of uncertainties, preventing more accurate predictions (6, 13). On these grounds, assessment of the influence of the different methods of pre-surgical tumor volume determination, their relationship with the underlying clinical and genetic biomarkers, or even with the efficacy of the neurosurgical resections, entail vital relevance to improve the accuracy of current OS and PFS predictions.

On these grounds, we aimed here to assess the influence of different methods of determination of the tumor size, tumor compartments, genetic profile, and some relevant clinical and neurosurgical variables, in the prediction of OS and PFS in primary glioblastomas. To this end, we investigated OS and PFS in a consecutive cohort of patients with primary IDH wild-type glioblastomas, selected as candidates for neurosurgical resection, followed by adjuvant chemo- and radiotherapy. Then, we compared measurements of the tumoral size and its compartments by the 2D diameter, the 3D ellipsoid, and the 3D semi-automatic (Smartbrush® software by Brainlab AG) methods, and correlated them with OS and PFS, considering also the profile of IDH and MGMT mutations, clinical variables as anatomical location or eloquence, and the extension of resection. We found high correlations among measurements of tumoral size by the three methods, with pre-operative FLAIR-T_2_ hyper-intensity areas and volumes providing the optimal neuroimaging biomarker to predict OS in primary glioblastoma patients, together with age ≥ 65 years and MGMT methylation.

## Materials and Methods

### Study Design

Our study was conducted in the period 2015–2019, with the consecutive inclusion of adult patients with newly diagnosed IDH wild-type glioblastomas (Grade IV WHO), treated in the Neurosurgery Department of the University Hospital La Paz, Madrid, Spain (https://www.comunidad.madrid/hospital/lapaz/). The protocol was approved by the institutional ethics committee, followed the rules of the Helsinki Declaration, and complied with the STROBE checklist (29).

All consecutive adult patients with suspected high-grade glioma, candidates for surgical resection were initially included. Thereafter, 44 patients were selected, with a definitive diagnosis of glioblastoma in the absence of IDH mutation. Inclusion criteria were:

- Pre-operative MRI, at least 72 h after treatment with dexamethasone (4 mg, every 8 h),
- Post-operative MRI, between 24 and 48 h after surgery, to assess the presence of tumor in contrast enhancement tissue (30, 31) and,
- Radiotherapy (2.0 grays Gy/day, 60 Gy total) and TMZ (75 mg/m^2^ oral daily) according to Stupp (32), followed by adjuvant TMZ (150–200 mg/m^2^ oral for 5 days during each 28-day cycle) with 6–12 cycles depending on the therapeutic response.

The exclusion criteria were patients who could not be offered the standard treatment (resective surgery followed by chemo-radiotherapy) because of very high surgical risk, or also those who refused surgical or chemo- and/or-radiotherapy treatments.

### MRI

Magnetic Resonance Imaging (MRI) was performed pre-operatively in a 3 Tesla magnet, (MAGNETOM Skyra, Siemens, Erlangen/Germany) and postoperatively at 1.5 Tesla (MAGNETOM Avanto, Siemens, Erlangen/Germany).

Pre-operative MRI included: Sagittal 3D-T_1w_-SPACE (TR: 650 ms, TE: 11 ms, and 1 mm isotropic resolution) before and after gadolinium (Dotarem® 0.2 mL/kg IV bolus infused at rate of 2 mL/s) administration, Axial and Coronal 2D-T_2w_-FSE (TR = 4,450 ms, TE: 83 ms, and slice thickness: 4 mm), Axial 2D Fluid-Attenuation Inversion Recovery (FLAIR) (TR = 12,000 ms, TE: 101 ms, and TI: 2,760).

Post-operative MRI included: Sagittal 3D-T_1w_-SPACE (TR: 600 ms, TE: 8 ms, and 1 mm isotropic resolution), Axial and Coronal 2D-T_2w_-FSE (TR: 4,000 ms, TE: 81 ms, and slice thickness: 5 mm), Axial 2D FLAIR (TR = 9,000 ms, TE: 92 ms, and TI: 2,500). Sagittal 3D-T_1w_-SPACE with contrast (TR: 450 ms, TE: 11 ms, and 1 mm isotropic resolution).

### Tumor Size Analysis

**Figure 1:**
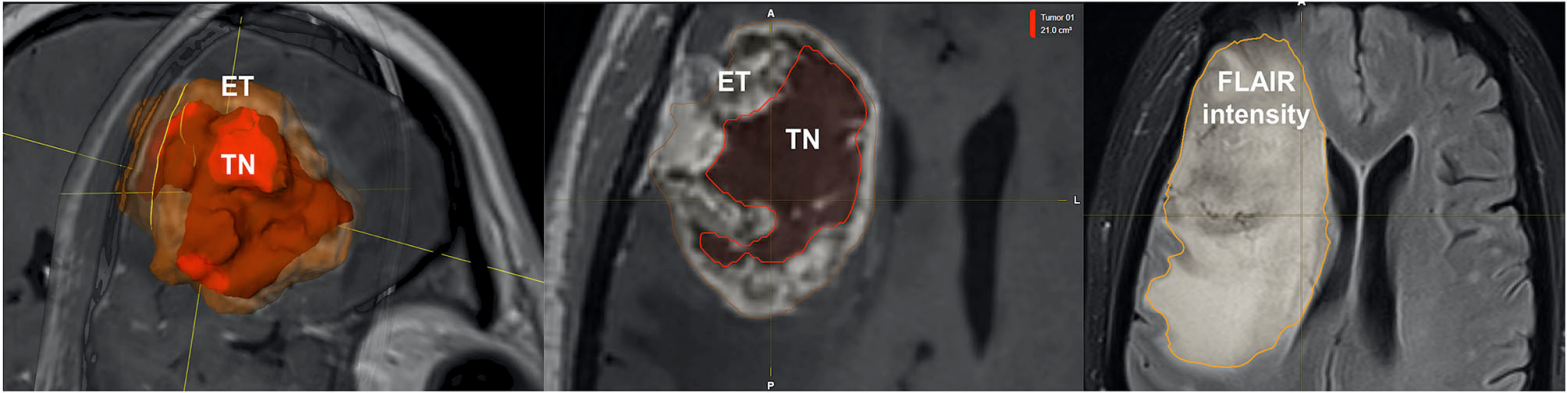
Representative volumetric parameters calculated by the semi-automatic method. Similar compartments were measured by the 2D and, 3D ellipsoid methods. Left: Enhancing tumor (ET), Center: Tumor necrosis (TN), Right: Fluid-attenuation inversion recovery (FLAIR intensity).

**Figure 2:**
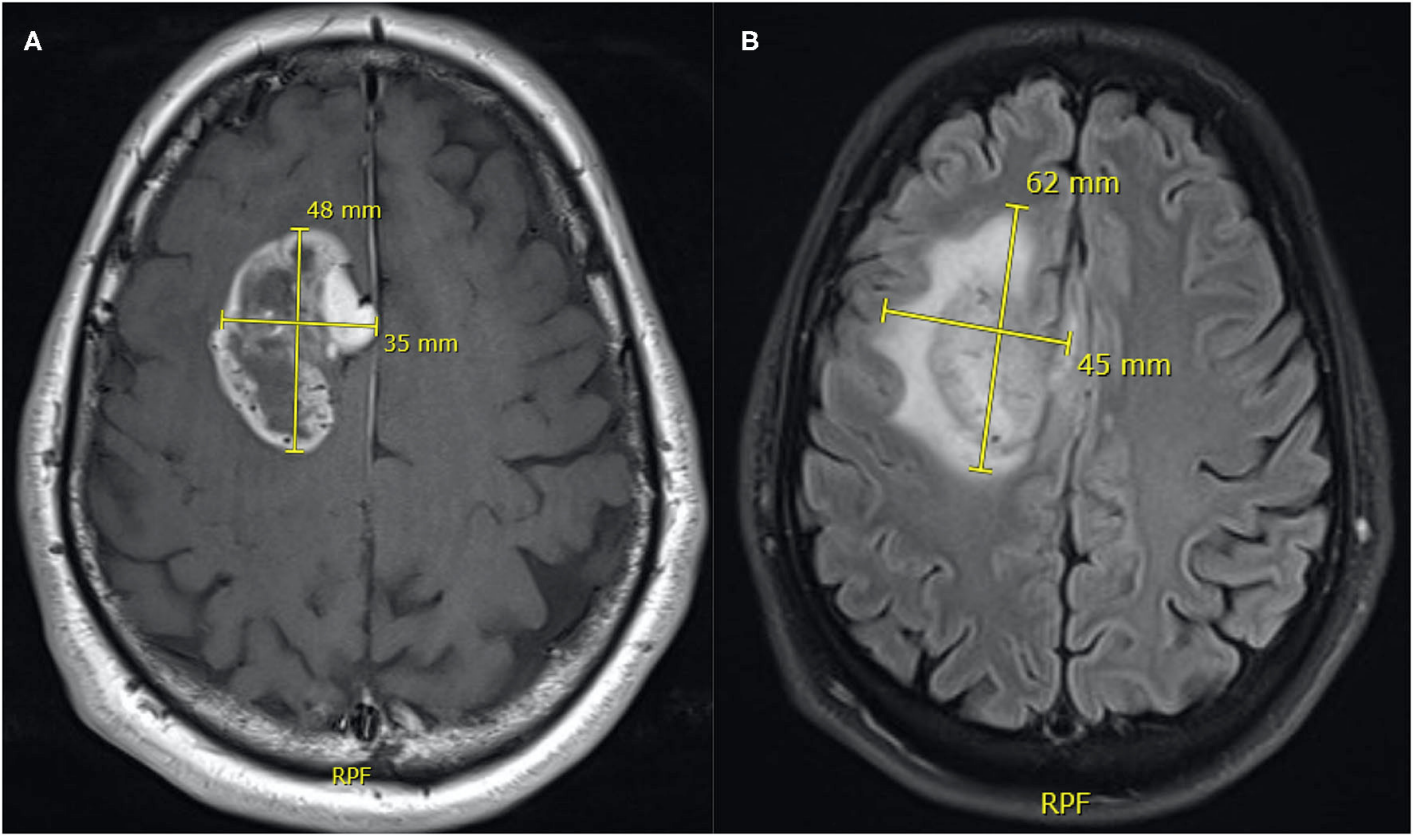
Representative measurements of tumor size **(A)** and FLAIR-T_2_ **(B)** area by the 2D orthogonal diameters method. The area of the tumor mass is the product of 48 × 35 mm = 1,680 mm^2^ and the FLAIR-T_2_ area is 62 × 45 mm = 2,790 mm^2^.

**Figure 3:**
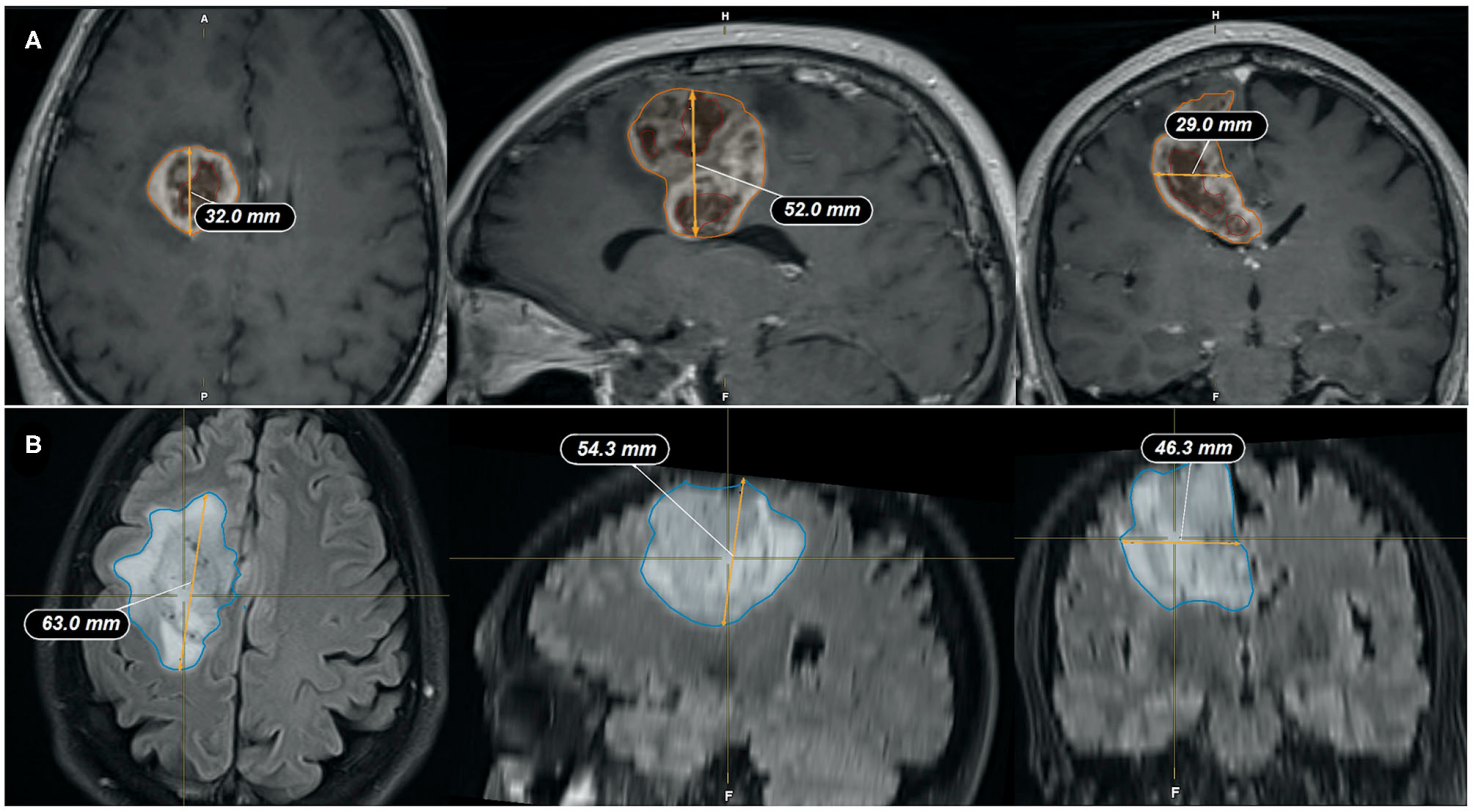
Representative measurements of the tumor size by the ellipsoid method. **(A)** Tumor mass volume is: (32 × 52 × 29 mm)/2 = 24.13 cm^3^; **(B)** FLAIR-T_2_ volume is: (63 × 54.3 × 46.3 mm)/2 = 79.19 cm^3^. Coronal (left), Sagital (center), and Axial (right) slices through the tumor.

**Figure 4:**
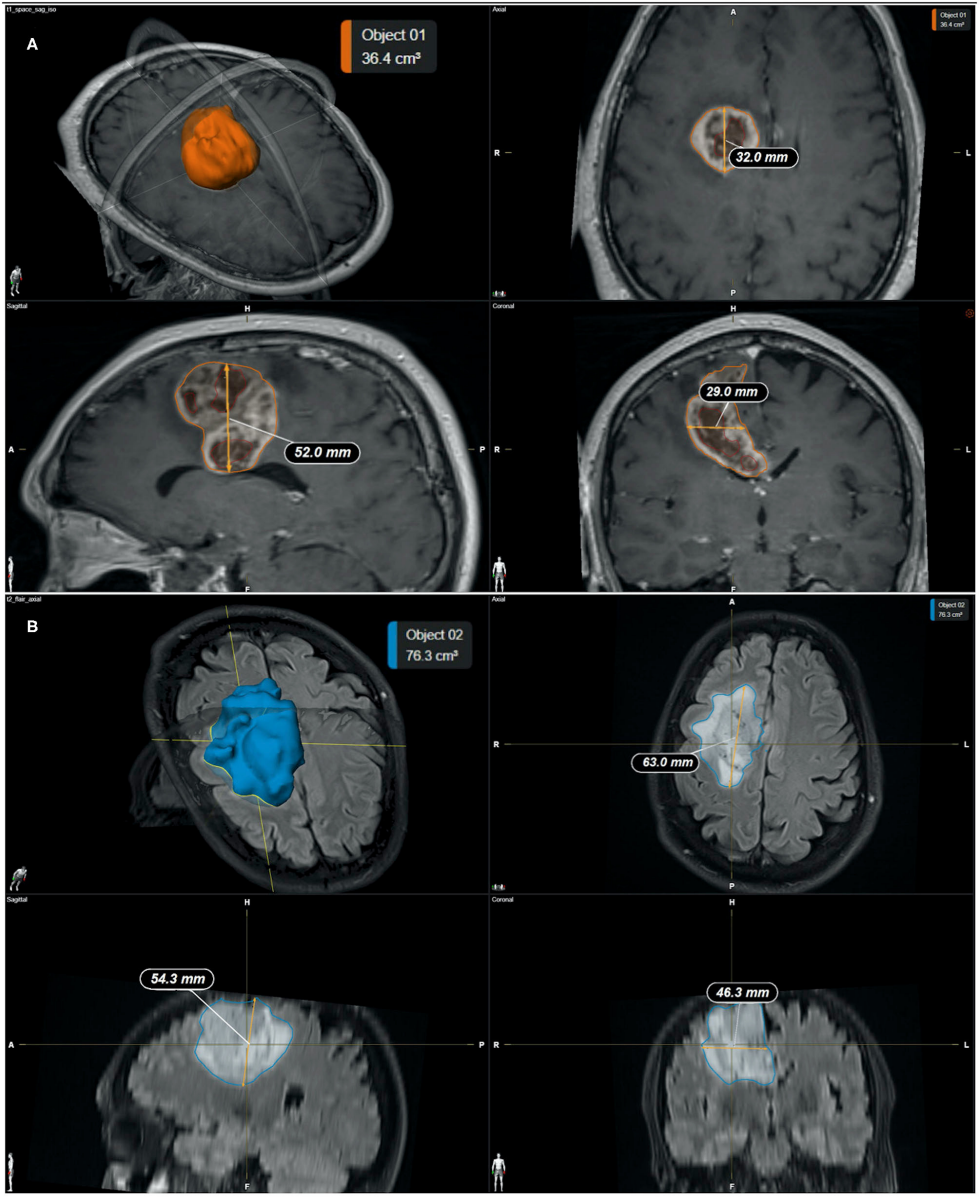
**(A)** Representative measurements of the tumor mass by the semi-automatic method (SmartBrush software—BrainLAB®). **(A)** Calculated tumor mass volume is 36.4 cm^3^; **(B)** Calculated FLAIR-T_2_ volume is 76.3 cm^3^. Individual panels indicate; 3D reconstruction (top left), Coronal (top right), Sagital (bottom left), and Axial (bottom right) slices through the tumor, respectively.

Quantitative measurements were performed by two neurosurgeons who jointly carried out the determinations. Representative pre-operative MRI measurements of tumor size measurements are illustrated in Figure 1, implementing either;

- 2D diameters method: as the sum of the products of perpendicular diameters of all contrast-enhancing lesions in T_1_, as recommended by RANO (23) (Figure 2),
- Orthogonal ellipsoid method: as the product of the longest perpendicular diameters in axial (a), sagittal (b), and coronal (c) sections divided by two (a × b × c/2), preferred to the formula 4/3 pi (a/2 × b/2 × c/2) for its easier and more reliable implementation (27, 33) (Figure 3); and
- Manually selecting the region of interest (ROI) using the Smartbrush tool of semi-automatic segmentation software (BrainLAB®, Munich Germany; Figure 4).

The following volumetric compartments, their definitions, and ratios were chosen, to be able to correlate with the OS estimates in previous studies (11–19):

- Enhancing tumor (ET) in the T_1_ sequence: As the tumor enhancement after gadolinium injection.
- Tumoral necrosis (TN): as the region of enhancing tumor which does not show enhancement after gadolinium administration. Non-necrotic cystic lesions were excluded.
- Tumor mass (TM): Sum of the enhancing tumor and necrosis.
- Hyperintensity in FLAIR-T_2_: as the region of hyper-intensity in FLAIR-T_2_ including the tumor mass and peritumoral area, including vasogenic edema and tumor infiltration.
- Residual tumor volume (RTV): Volume of enhancement that results from the post-contrast and pre-contrast subtraction in the T_1_ sequence, reflecting the residual “enhancing tumor.”
- Extent of resection (EOR): as the result of: [(initial enhancing tumor) – (the remaining enhancing tumor after resection)]/(initial enhancing tumor) expressed as a percentage.
- Necrosis/Tumor mass ratio (NTR).
- FLAIR-T_2_/Tumor mass ratio (FTR).

### Immunohistochemistry and Genetic Assessment

Anatomopathological analysis of biopsies obtained during neurosurgery included the immunohistochemical detection of the IDH (isocitrate dehydrogenase R132H) mutation. In negative cases, with high suspicion of IDH mutation no-R132H, a genetic study was performed by pyrosequencing (34). All cases underwent analysis of the methylation status of the O^6^ methylguanine-DNA methyltransferase (MGMT) promoter using the MethyLigh PCR reaction (35).

### Statistics

Univariate statistical analyses included analysis of means, median and standard deviation. Bivariate analysis were performed according of the type of variable; Pearson and Spearman's correlation coefficient to compare among volumetric and diameter measurements by three methods, Mann–Whitney *U*-test for volumes according to the state of MGMT methylation, with log-rank test used for time-event analyzes and survival analysis using the Kaplan–Meier method. Finally, the multivariate analysis was performed using Cox regression test to assess the independence of possible prognostic factors in overall and progression free survival for the three measurement techniques. The analysis was conducted using Microsoft Excel 2016 and the statistical package Stata version 14 (StataCorp LLC, Texas, USA) as implemented in a personal PC operating under the MacOS environment.

## Results

Table 1 summarizes the patient cohort and tumor characteristics of our study. A total of 44 patients met the inclusion criteria. Mean age was 58.15 ± 11.36 (range 35–81 years), with a male/female ratio of 1.9/1, reflecting masculine predominance (2). The median of Karnofsky index (37) was 90 (range 70–100) with the frontal location most frequently found 13/44 (29.5%) and the right/left distribution of 0.7/1. The percentage tumors in eloquent and near-eloquent areas (36) were 9.1 and 68.2%, respectively. Most frequent clinical presentations included; headache (40%), cognitive deficit (31.1%), and motor deficit (26.7%). All patients were IDH negative and the percentage of the methylation of the MGMT promoter was 38.6%. Six cycles of chemotherapy each 28 days with TMZ was completed in 63.6% of patients. Re-operation was performed in 15.6% of them. A second line of chemotherapy was administered in 28.9%, with Fotemustine® (22.7%) and Lomustine® (6.8%) as the most used drugs. Median percentage of tumor resection was 95.9% (range 62.3–100%).

**Table 1:** Clinical, genetic, neurosurgical, and chemotherapy variables of the patient cohort and associated overall survival, and progression free survivals.

**Parameter**		***n* = 44**
Age	Mean (years) ± SD (range)	58.2 ± 11.4 (35–81)
Gender	Male	29 (65.9%)
	Female	15 (34.1%)
Comorbidity^a^		26 (59.1%)
Karnofsky Scale	Median (range)	90 (70–100)
Hemisphere	Right	26 (59.1%)
	Left	18 (40.9%)
Eloquency^b^	Not eloquent ratio	10/44 (22.7%)
	Near-eloquent ratio	30/44 (68.2%)
	Eloquent ratio	4/44 (9.1%)
Location	Frontal	13 (29.5%)
	Temporal	11 (25%)
	Parietal	11 (25%)
	Occipital	9 (20.5%)
	Deep	0 (0%)
	Multicentric	4 (9.1%)
MGMT status	Methylated	17 (38.6%)
	Non-methylated	27 (61.4%)
IDH wild-type		100%
Extent of Resection	%, Median (Q1–Q3)	95.9 (89.03–100)
Stupp protocol^c^		100%
Chemotherapy with TMZ	Median of cycles (Q1–Q3)	6 (3–12)
Second line of chemotherapy^d^		10 (22.7%)
Progression free survival	Median (months), 95% CI	7 (5–9)
Overall survival	Median (months), 95% CI	16 (11–19)

a *Arterial hypertension, diabetes mellitus, and/or pulmonary, renal, cardiac, oncologic, or any severe disease*,

b *Eloquency according to Sawaya et al. (36); TMZ, Temozolamide; MGMT, O^6^-methylguanin-DNA-methyltransferase*;

c *Stupp protocol, Radiotherapy (2.0 grays [Gy]/day; total dose, 60 Gy), and temozolomide (75 mg/m^2^ orally daily) (32)*;

d *Lomusitne or Fotemustine; Q1-Q3 first and third quartiles (interquartile range); CI, confidence interval*.

### Tumor Size and Compartmental Correlation Analysis: Diameters, Ellipsoid, and Semi-automatic Methods

**Figure 5:**
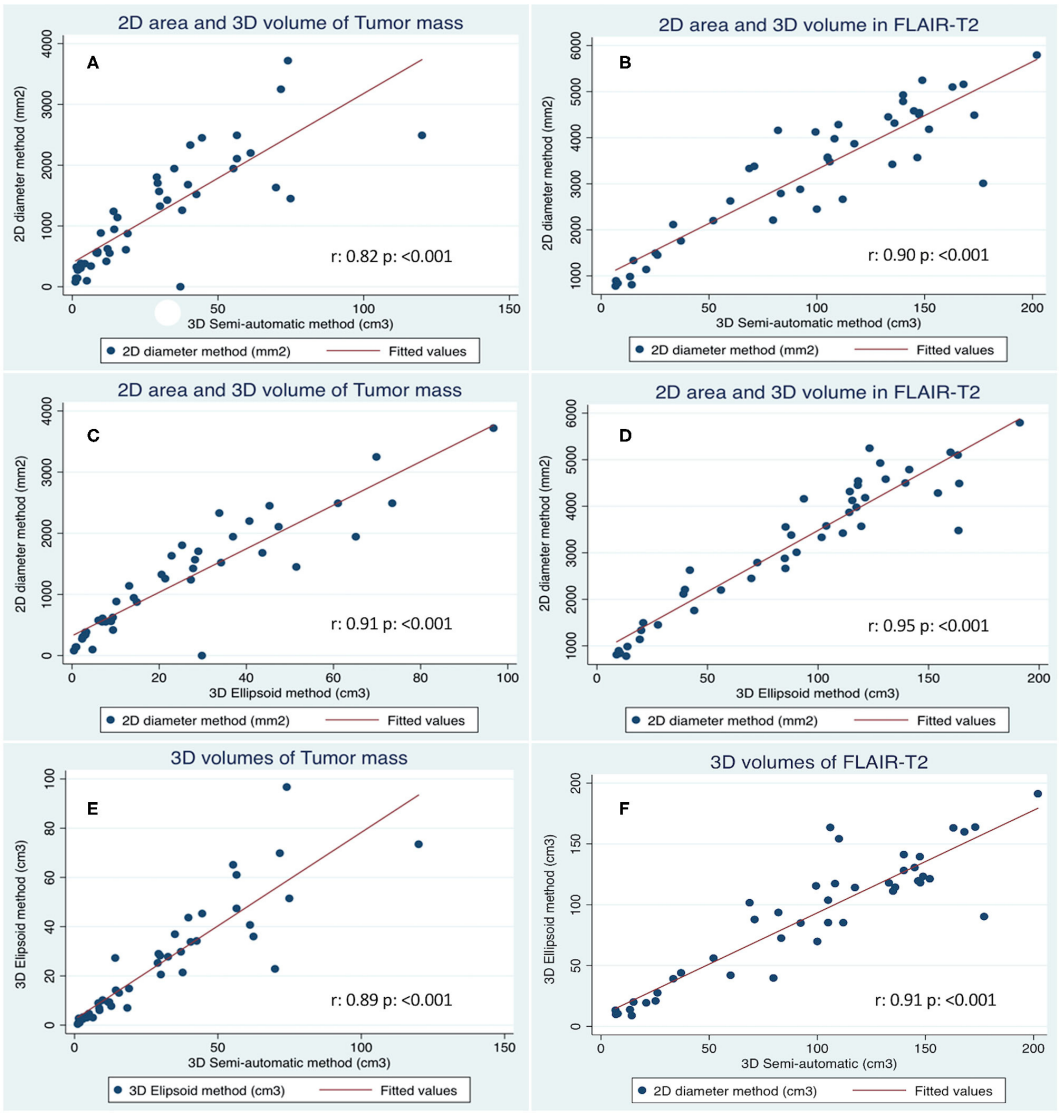
Correlation of tumor size measurements as performed with the three methods. **(A)** Tumor mass calculated with the 2D diameter vs. 3D Semi-automatic methods. **(B)** FLAIR-T_2_ area and volume calculated with 2D diameter vs. 3D semi-automatic methods. **(C)** Tumor mass calculated with the 2D diameter vs. 3D Ellipsoid method. **(D)** FLAIR-T_2_ area and volume calculated with 2D diameter vs. 3D Ellipsoid method. **(E)** Tumor mass calculated with the 3D Ellipsoid vs. 3D Semi-automatic method. **(F)** FLAIR-T_2_ volumes calculated with 3D Ellipsoid vs. 3D Semi-automatic methods.

Table 2 summarizes measurements of different tumor compartmental areas and volumes with the three methods investigated, while Figure 5 provides the correlations within them. Most of the compartmental volumes determined by the 3D ellipsoid and automatic segmentation methods were similar, revealing consistency. Similarly, using the sum of the products of major perpendicular diameters, the mean of tumor area had a high correlation with the tumor mass volume measured by the ellipsoid (*r* = 0.91, *p* < 0.001), and semi-automatic segmentation (*r* = 0.82, *p* < 0.001) methods. The mean FLAIR-T_2_ area also highly correlated with the FLAIR-T_2_ volume by the ellipsoid method (*r* = 0.95, *p* < 0.001), and the semi-automatic segmentation (*r* = 0.90, *p* < 0.001) methods (Figure 5). The mean of the enhancing tumor and of the necrosis volumes were only assessed by the semi-automatic segmentation method, since the irregular morphology hampered the use of the 2D diameter method and the ellipsoid formula.

**Table 2:** Tumor and compartment volumes as calculated by 2D diameter, ellipsoid, and semi-automatic segmentation methods.

**Method**	**Compartment**	**Value (units)**
2D diameter	Area of the tumor mass^a^	1,203 ± 911 (80–3,720) mm^2^
	Area of hyperintensity in FLAIR-T_2_	3,210 ± 1,422 (780–5,795) mm^2^
	FLAIR-T_2_/tumor ratio^c^	2.91 ± 3.48 (1.2–17.9)
3D Ellipsoid	Tumoral mass	24.9 ± 23.1 (0.4–96.7) cm^3^
	FLAIR-T_2_ volume	89.7 ± 51.2 (8.9–191) cm^3^
	FLAIR-T_2_/tumor ratio^c^	4.14 (2.6–7.15)
3D semi-automatic	Enhancing tumor^b^ (T1+Gd)	14.2 ± 12.2 (0.8–47) cm^3^
	Necrosis^b^	15.6 ± 18.3 (0–73) cm^3^
	Tumoral mass	29.8 ± 26.9 (1.1–120) cm^3^
	FLAIR-T_2_ volume	95.7 ± 55.2 (6.7–202) cm^3^
	FLAIR-T_2_/tumor ratio^c^	3.39 (2.32–5.5)
	Necrosis/tumor ratio^b, c^	0.44 (0.26–0.59)

*Results are expressed as mean ± SD with range of measured values in parenthesis*.

a *Necrosis + enhancing tumor in T1+Gd*.

b *Only measurable by Semi-automatic method*.

c *Q1 and Q3 indicate the first and third quartiles*.

We found a high correlation between the means of the tumor mass volume (Figure 5E, *r* = 0.89, *p* < 0.001) and between the means of the FLAIR-T_2_ volumes (Figure 5F, *r* = 0.91, *p* < 0.001) by both methods. The correlation between the means of the FLAIR-T_2_/tumor ratio were not as high as the primary measurements, between the 2D diameter and the ellipsoid method was (*r* = 0.69, *p* < 0.001), between ellipsoid and semi-automatic methods was (*r* = 0.84, *p* < 0.001) and between 2D diameter and semi-automatic method was (*r* = 0.56, *p* < 0.001). The necrosis/tumor ratio could only be obtained by the semi-automatic method and therefore could not be correlated with other methods.

### Survival Analysis

**Figure 6:**
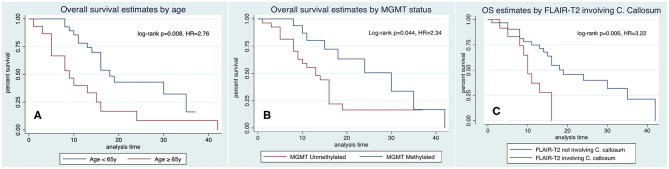
Kaplan–Meyer analysis of OS as predicted by age **(A)**, methylation status of the MGMT promoter **(B)**, and hyperintensity in FLAIR-T_2_ involving the corpus callosum **(C)**.

Figure 6 illustrates Kaplan–Meier profiles of OS depending on the age of the patients and methylation status of the tumors. Median OS of the investigated cohort was 16 ± 9.1 months (range 1–42 months). Gender, Karnofsky status, location of the tumor, eloquence and extent of tumor resection, had no impact on OS, although tumors with a extent of resection higher than 98% showed a tendency to greater survival, almost reaching statistical significance (18 ± 2.6 vs. 14 ± 4.2 months, *p* = 0.497). The following features merit further comments.

*Age*. Elderly were associated with a higher mortality in the bivariate analysis, being 18 ± 1.6 months in patients < 65 years, and 9 ± 2.4 months in patients ≥ 65 years (Figure 6A, HR 2.76, *p* = 0.008).

*MGMT methylation* was associated with longer OS, 30 ± 5.2 months in methylated vs. 13 ± 2.5 months in non-methylated (Figure 6B, HR = 2.34, *p* = 0.044). Necrosis, tumor mass, and FLAIR-T_2_ volumes in non-methylated MGMT tumors were larger than in methylated MGMT tumors: 18.7 vs. 10.7 cm^3^, 33.9 vs. 23.3 cm^3^, and 104.3 vs. 82.1 cm^3^, respectively, without statistical significance (Mann–Whitney *U-*test *p* = 0.161, 0.204, and 0.195, respectively).

*Hyperintensity in FLAIR-T*_2_ *involving the corpus callosum* had a significant impact in OS, 10 ± 1.08 months when there was involvement of the corpus callosum vs. 19 ± 3.7 months when there was no involvement of the corpus callosum (Figure 6C, HR = 3.22, *p* = 0.006).

*Gadolinium enhancing tumor, necrosis, and the tumor mass volumes* calculated by the 3D ellipsoid and 3D semi-automatic methods did not have any impact on OS and PFS, and *the tumor mass area*, calculated with the 2D diameter method, was not associated with OS or PFS either.

**Figure 7:**
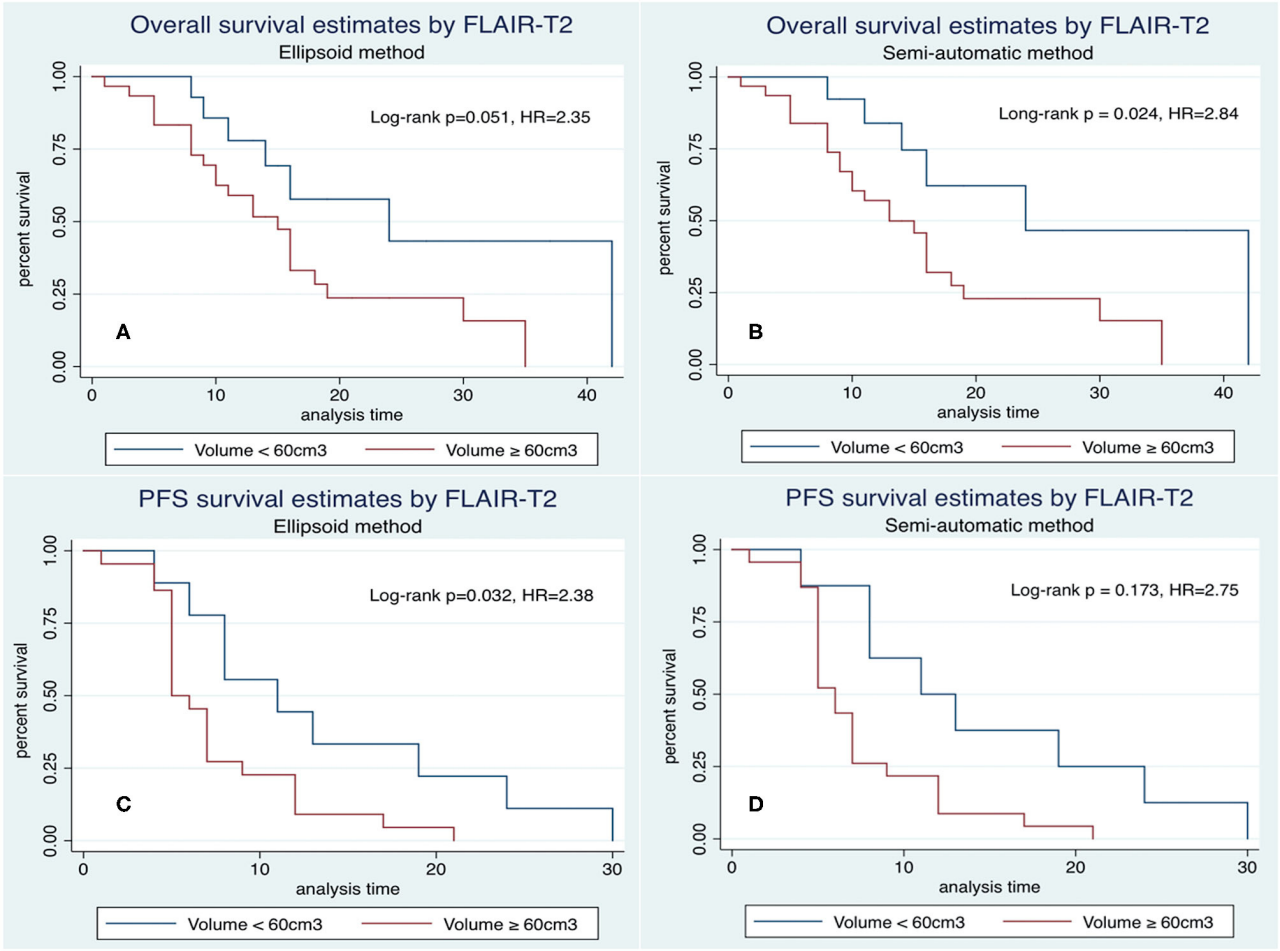
Kaplan–Meier representation of overall survival **(A,B)** and progression-free survival **(C,D)** related to pre-operative FLAIR-T_2_ volume ≥ 60 cm^3^ by 3D ellipsoid **(A–C)** and semi-automatic segmentation **(B–D)** methods.

Figure 7 and Tables 3–5, summarizes Kaplan–Meier tests of OS and PFS with pre-operative FLAIR-T_2_ volume measurements by the 3D ellipsoid, and semiautomatic segmentation methods, respectively.

**Table 3:** Multivariate Cox Regression analysis with tumor size as determined by the 2D diameter method.

**Variable**	**Unadjusted**	**95% CI**	***p*-value**	**Adjusted**	**95% CI**	***p*-value**
	**HR**			**HR**		
Age ≥ 65 years	2.76	1.30–5.84	0.008	2.85	1.26–6.41	0.011
MGMT Unmethylated	2.34	1.02–5.38	0.044	2.73	1.01–7.47	0.050
FLAIR-T_2_ ≥ 2,000 mm^2^	3.06	1.04–8.97	0.041	3.52	1.11–11.2	0.027
FTR^a^ ≥ 5	0.97	0.41–2.34	0.954	1.84	0.68–5.02	0.229

a *FLAIR-T_2_/tumor ratio; HR, Hazard ratio; CI, confidence interval*.

**Table 4:** Multivariate Cox Regression analysis with tumor volumetry as determined by the ellipsoid method.

**Variable**	**Unadjusted**	**95% CI**	***p*-value**	**Adjusted**	**95% CI**	***p*-value**
	**HR**			**HR**		
Age ≥ 65 years	2.76	1.30–5.84	0.008	3.27	1.45–7.38	0.004
MGMT Unmethylated	2.34	1.02–5.38	0.044	2.25	0.98–5.43	0.051
FLAIR-T_2_ ≥ 60 cm^3^	2.35	0.95–5.84	0.051	2.83	1.08–7.44	0.034
FTR^a^ ≥ 5	1.49	0.69–3.19	0.302	1.43	0.64–3.19	0.321

a *FLAIR-T_2_/tumor ratio; HR, Hazard ratio; CI, confidence interval*.

**Table 5:** Multivariate Cox Regression analysis with tumor volumetry as determined with the semi-automatic method.

**Variable**	**Unadjusted**	**95% CI**	***p*-value**	**Adjusted**	**95% CI**	***p*-value**
	**HR**			**HR**		
Age ≥ 65 years	2.76	1.30–5.84	0.008	3.39	1.51–7.61	0.003
MGMT Unmethylated	2.34	1.02–5.38	0.044	2.53	1.03–6.57	0.046
FLAIR-T_2_ ≥ 60 cm^3^	2.84	1.07–7.55	0.024	3.93	1.23–10.2	0.018
FTR^a^ ≥ 5	1.02	0.45–2.34	0.956	1.62	0.62–4.18	0.321

a *FLAIR-T_2_/tumor ratio; HR, Hazard ratio; CI, confidence interval*.

*FLAIR-T*_2_ *volume* ≥ *60 cm*^3^ by the semi-automatic method was associated with shorter OS, with median survival times of 13 ± 1.8 m (≥60 cm^3^) vs. 24 ± 6.5 m (<60 cm^3^), respectively (Figure 7B, HR = 2.84, *p* = 0.024). A similar impact was observed with the 3D ellipsoid method, with the median survival ranging from 15 ± 1.8 (≥60 cm^3^) to 24 ± 5.8 m (<60 cm^3^), (Figure 7A, HR = 2.35, *p* = 0.051), reaching closely statistical significance. The FLAIR-T_2_ volume ≥ 60 cm^3^ had also impact in progression-free survival by both 3D methods (Figures 7C,D); *FLAIR-T*_2_ *area* ≥ *2,000 mm*^2^ as determined by the 2D diameter method was also associated with shorter OS (HR = 3.06, *p* = 0.041).

*Necrosis/Tumor ratio* had no impact on survival (*p* = 0.798). *The FLAIR-T*_2_*/tumor ratio* by the semi-automatic method had a negative impact on survival (*p* = 0.008) without detecting such an association with the 3D ellipsoid and 2D diameter methods.

### Multivariate Analysis

Finally, we implemented a multivariate analysis (Cox Regression analysis) strategy to identify the independent predictors of OS among the demographic, imaging, and genetic variables that were significant in the bivariate analysis. We carried out the analysis individualizing the results for each method (Tables 3–5). The following variables were involved; age ≥ 65 years, MGMT methylation status, FLAIR-T_2_ abnormality ≥ 60 cm^3^ or ≥ 2,000 mm^3^ and FLAIR-T_2_/tumor ratio ≥ 5 for all methods. The variable Hyperintensity in FLAIR-T_2_ involving the corpus callosum was excluded because did not provide more significance to the multivariate models due to the close association with the variable FLAIR-T_2_ volume > 60 cm^3^ (Fisher's exact test *p* = 0.009). We found that independence of the variables FLAIR-T_2_ > 60 cm^3^, age > 65 years, as well as the MGMT methylation status, remained significant with a similar impact on OS than in the bivariate analysis.

## Discussion

Our pilot study investigates the prognostic value of three different methods of pre-surgical tumor size measurement by MRI, combined with relevant clinical and genetic information, as OS and PFS predictors. Briefly, we selected a homogeneous cohort of patients harboring primary glioblastomas (IDH wild type), who were candidates for resective surgery and adjuvant chemo-radiotherapy. Tumor sizes and compartments were determined pre-operatively using the 2D diameter method recommended by the RANO (23, 24), the 3D ellipsoid formula (25, 26) and the semi-automatic 3D segmentation (33) methods. Clinical and genetic variables, including gender, age, expression of IDH mutation, and MGMT status, were investigated additionally.

In general, our results confirm the correlation of elderly patients with shorter OS, with the cut-off point in terms of survival prediction in 65 years, as well as the methylated status of the MGMT promoter with longer OS, two well-known variables normally associated with OS and PFS (6, 11). In addition, incomplete neurosurgical resections, considered as important negative survival markers (4, 38–40), depicted in the present study, only a trend to shorter OS without statistical significance, a finding probably related to the relatively small sample of the present patient cohort.

As far as pre-operative measurement of tumor size is concerned, the three methods employed in this study presented a high correlation (41, 42). Despite the semi-automatic segmentation method is currently considered the most accurate, because of its high adaptability to irregular morphologies, and the possibility to determine, separately, the different compartmental volumes (27, 43), its advantages in terms of prognosis over the 2D diameter and the 3D ellipsoid methods, did not reach statistical significance, even in measurements of tumor size in pre-operative images. This may be caused, at least in part, by the relatively homogeneous tumor morphology in the pre-operative period. However, post-surgical images show limits and contours much more difficult to be silhouetted, and consequently, the semi-automatic method is the only one that can provide insight of the actual volume of the post-surgical residual tumor volume.

Among the areas, volumes and ratios investigated, including necrosis, gadolinium enhancement in T_1_, tumor mass, necrosis/tumor, and FLAIR-T_2_/tumor ratios, only FLAIR-T_2_ hyper-intensity had an important impact in OS and PFS, with the three methods of tumor size measurement used. Hyper-intensity in FLAIR-T_2_ surrounding tumor mass includes a mixture of peritumoral reactive edema and tumor cells with variable density, as has been previously characterized (44–46). Present results confirm that, the higher the volume of the FLAIR-T_2_ image, the worse the prognosis is. However, there is some controversy in this respect, since some studies have shown correlations between FLAIR-T_2_ hyper-intensities with longer OS (13–18, 40, 47), while others have not been able to prove it (12, 13, 48, 49). Many factors, including the non-exclusion of cystic cavities, the influence of corticosteroid treatment, and the inclusion of both, IDH-mutated, and IDH wild-type tumors, may further underlie these discrepancies.

Additionally, some studies have reported associations with survival of the necrosis volume, enhancement tumor volume, and the tumor/necrosis ratio (13, 18, 48). Our study could not confirm these, either in either bivariate or multivariate analyses. However, we observed a trend indicating that the volume of FLAIR-T_2_ hyper-intensity, tumor mass, and necrosis, were greater in the tumors with non-methylated MGMT promoter (50). The lower survival of this glioblastoma subgroup is clearly described by its greater resistance to alkylating chemotherapeutic agents, but it could also involve a higher rate of tumor growth, with higher volumes being observed in all measurements when diagnosing the disease, and before any treatment prescribed. Future studies with a larger sample size could contribute to clarify this finding.

Summarizing, our study shows that the size of FLAIR-T_2_ hyper-intensity, as measured by the three methods, presents a similar significant impact in the OS and PFS under both, bivariate and multivariate analyses. The larger the FLAIR-T_2_ hyper-intensity volume, the shorter OS and PFS predictions. On these basis, surgical resection of the largest part of the FLAIR-T_2_ hyperintensity tissue might determine a better prognosis, in agreement with previous studies (40, 51, 52). Additionally we show, that the 3D semi-automatic method, more complex and time consuming than the other alternatives (22, 53), ends up providing similar pre-operative tumor volumetry results, without any preponderance in terms of survival. Consequently, our study supports the use of the 2D diameter and 3D ellipsoid methods, to estimate FLAIR-T_2_ hyper-intensity areas and volumes, revealed here as parameters with an important impact in OS and PFS predictions.

### Limitations

Acknowledged limitations of this pilot study relate to the single center implementation and the reduced size of the patient cohort. Thus, increasing the number of patients examined and extension to multicenter assays would constitute immediate priorities to validate the present observations.

## Conclusions

The present study reveals that pre-operative tumor size estimations in primary glioblastoma, as determined by the 2D diameter and 3D ellipsoid methods, provide similar results to the 3D semi-automatic segmentation method. The three methods reveal that FLAIR-T_2_ hyperintensity areas and volumes, provide independent factors closely associated with OS and PFS predictions. The larger the FLAIR-T_2_ hyper-intensity volume, the worse OS and PFS prediction. In addition, we emphasize the negative impact of age ≥ 65 years and non-methylation of the MGMT promoter in survival predictions.

## Data Availability Statement

The raw data supporting the conclusions of this article will be made available by the authors, without undue reservation.

## Ethics Statement

The studies involving human participants were reviewed and approved by Ethics Commitee University Hospital La Paz, Madrid. The patients/participants provided their written informed consent to participate in this study.

## Author Contributions

AP performed most of the surgeries, collected and integrated the patient data, applied the different volumetric methods to the image database, provided the univariate and multivariate statistical analyses, and wrote the first draft of the manuscript. CV collected and integrated the patient and imaging data, implemented the MRI volumetric methods, and contributed to the first draft of the manuscript. AB, CU, and AR contributed in the radiologic assessment of pre- and post-operative MRI and validated the volumetric measurements. MG performed many of the surgeries, validated demographic and histopathological assessments, and contributed to the final version of the manuscript. SC and JR integrated all clinical, neurosurgical, and imaging information and accomplished the writing of the final version, with all authors commenting. All authors contributed to the article and approved the submitted version.

## Conflict of Interest

The authors declare that the research was conducted in the absence of any commercial or financial relationships that could be construed as a potential conflict of interest.

## Abbreviations

CI: Confidence Interval
d: days
EOR: Extent of resection
ET: Enhancing tumor
FLAIR: Fluid-attenuation inversion recovery
FTR: FLAIR-T_2_/Tumor mass ratio
HR: Hazard ratio (Cox method)
IDH: Isocitrate dehydrogenase
m: months
MGMT: O^6^-methylguanin-DNA-methyltransferase
MRI: Magnetic resonance imaging
NTR: Necrosis/Tumor mass ratio
PFS: Progression-free survival
ROI: region of interest
RTV: Residual tumor volume
TM: Tumor mass
TMZ: Temozolamide
TN: Tumoral necrosis
y: years.

## References

[1] Deorah S Lynch CF Sibenaller ZA Ryken TC Trends in brain cancer incidence and survival in the United States: surveillance, epidemiology, and end results program, 1973 to 2001. Neurosurg Focus. (2006) 20:E1 10.3171/foc.2006.20.4.E1 16709014

[2] Ostrom QT Gittleman H Fulop J Liu M Blanda R Kromer C CBTRUS statistical report: primary brain and central nervous system tumors diagnosed in the United States in 2008-2012. Neuro Oncol. (2015) 17(Suppl 4):iv1–62. 10.1093/neuonc/nov189 26511214 PMC4623240

[3] Hou LC Veeravagu A Hsu AR Tse VCK Recurrent glioblastoma multiforme: a review of natural history and management options. Neurosurg Focus. (2006) 20:E5 10.3171/foc.2006.20.4.2 16709036

[4] Stupp R Hegi ME Mason WP van den Bent MJ Taphoorn MJB Janzer RC Effects of radiotherapy with concomitant and adjuvant temozolomide versus radiotherapy alone on survival in glioblastoma in a randomised phase III study: 5-year analysis of the EORTC-NCIC trial. Lancet Oncol. (2009) 10:459–66. 10.1016/S1470-2045(09)70025-7 19269895

[5] Ohgaki H Kleihues P The definition of primary and secondary glioblastoma. Clin Cancer Res. (2013) 19:764–72. 10.1158/1078-0432.CCR-12-3002 23209033

[6] Louis DN Perry A Reifenberger G von Deimling A Figarella-Branger D Cavenee WK The 2016 World Health Organization Classification of Tumors of the Central Nervous System: a summary. Acta Neuropathol. (2016) 131:803–20. 10.1007/s00401-016-1545-1 27157931

[7] Nobusawa S Watanabe T Kleihues P Ohgaki H IDH1 mutations as molecular signature and predictive factor of secondary glioblastomas. Clin Cancer Res. (2009) 15:6002–7. 10.1158/1078-0432.CCR-09-0715 19755387

[8] Esteller M Garcia-Foncillas J Andion E Goodman SN Hidalgo OF Vanaclocha V Inactivation of the DNA-repair gene MGMT and the clinical response of gliomas to alkylating agents. N Engl J Med. (2000) 343:1350–4. 10.1056/NEJM200011093431901 11070098

[9] Hegi ME Diserens A-C Gorlia T Hamou M-F de Tribolet N Weller M MGMT gene silencing and benefit from temozolomide in glioblastoma. N Engl J Med. (2005) 352:997–1003. 10.1056/NEJMoa043331 15758010

[10] Gandía-González ML Cerdán S Barrios L López-Larrubia P Feijoó PG Palpan A Jr Assessment of overall survival in glioma patients as predicted by metabolomic criteria. Front Oncol. (2019) 9:328 10.3389/fonc.2019.00328 31134147 PMC6524167

[11] Lacroix M Abi-Said D Fourney DR Gokaslan ZL Shi W DeMonte F A multivariate analysis of 416 patients with glioblastoma multiforme: prognosis, extent of resection, and survival. J Neurosurg. (2001) 95:190–8. 10.3171/jns.2001.95.2.0190 11780887

[12] Iliadis G Kotoula V Chatzisotiriou A Televantou D Eleftheraki AG Lambaki S Volumetric and MGMT parameters in glioblastoma patients: survival analysis. BMC Cancer. (2012) 12:3 10.1186/1471-2407-12-3 22214427 PMC3264493

[13] Henker C Kriesen T Glass Ä Schneider B Piek J Volumetric quantification of glioblastoma: experiences with different measurement techniques and impact on survival. J Neurooncol. (2017) 135:391–402. 10.1007/s11060-017-2587-5 28755324

[14] Hammoud MA Sawaya R Shi W Thall PF Leeds NE Prognostic significance of preoperative MRI scans in glioblastoma multiforme. J Neurooncol. (1996) 27:65–73. 10.1007/BF00146086 8699228

[15] Pope WB Sayre J Perlina A Villablanca JP Mischel PS Cloughesy TF MR imaging correlates of survival in patients with high-grade gliomas. AJNR Am J Neuroradiol. (2005) 26:2466–74. 16286386 PMC7976216

[16] Li W-B Tang K Chen Q Li S Qiu X-G Li S-W MRI manifestions correlate with survival of glioblastoma multiforme patients. Cancer Biol Med. (2012) 9:120–3. 10.3969/j.issn.2095-3941.2012.02.007 23691466 PMC3643659

[17] Schoenegger K Oberndorfer S Wuschitz B Struhal W Hainfellner J Prayer D Peritumoral edema on MRI at initial diagnosis: an independent prognostic factor for glioblastoma? Eur J Neurol. (2009) 16:874–8. 10.1111/j.1468-1331.2009.02613.x 19473360

[18] Wu C-X Lin G-S Lin Z-X Zhang J-D Chen L Liu S-Y Peritumoral edema on magnetic resonance imaging predicts a poor clinical outcome in malignant glioma. Oncol Lett. (2015) 10:2769–76. 10.3892/ol.2015.3639 26722240 PMC4665258

[19] Pierallini A Bonamini M Osti MF Pantano P Palmeggiani F Santoro A Supratentorial glioblastoma: neuroradiological findings and survival after surgery and radiotherapy. Neuroradiology. (1996) 38(Suppl 1):S26–30. 10.1007/BF02278114 8811675

[20] Macdonald DR Cascino TL Schold SC Cairncross JG Response criteria for phase II studies of supratentorial malignant glioma. J Clin Oncol. (1990) 8:1277–80. 10.1200/JCO.1990.8.7.1277 2358840

[21] Wen PY Macdonald DR Reardon DA Cloughesy TF Sorensen AG Galanis E Updated response assessment criteria for high-grade gliomas: response assessment in neuro-oncology working group. J Clin Oncol. (2010) 28:1963–72. 10.1200/JCO.2009.26.3541 20231676

[22] Dempsey MF Condon BR Hadley DM Measurement of tumor “size” in recurrent malignant glioma: 1D, 2D, or 3D? AJNR Am J Neuroradiol. (2005) 26:770–6. 15814919 PMC7977136

[23] Wen PY Chang SM Van den Bent MJ Vogelbaum MA Macdonald DR Lee EQ Response assessment in neuro-oncology clinical trials. J Clin Oncol. (2017) 35:2439–49. 10.1200/JCO.2017.72.7511 28640707 PMC5516482

[24] Ellingson BM Wen PY Cloughesy TF Modified criteria for radiographic response assessment in glioblastoma clinical trials. Neurotherapeutics. (2017) 14:307–20. 10.1007/s13311-016-0507-6 28108885 PMC5398984

[25] Opalak CF Parry M Rock AK Sima AP Carr MT Chandra V Comparison of ABC/2 estimation and a volumetric computerized method for measurement of meningiomas using magnetic resonance imaging. J Neurooncol. (2019) 144:275–82. 10.1007/s11060-019-03205-z 31401721

[26] Leu S Boulay J-L Thommen S Bucher HC Stippich C Mariani L Preoperative two-dimensional size of glioblastoma is associated with patient survival. World Neurosurg. (2018) 115:e448–63. 10.1016/j.wneu.2018.04.067 29678715

[27] Sorensen AG Patel S Harmath C Bridges S Synnott J Sievers A Comparison of diameter and perimeter methods for tumor volume calculation. J Clin Oncol. (2001) 19:551–7. 10.1200/JCO.2001.19.2.551 11208850

[28] Czarnek N Clark K Peters KB Mazurowski MA Algorithmic three-dimensional analysis of tumor shape in MRI improves prognosis of survival in glioblastoma: a multi-institutional study. J Neurooncol. (2017) 132:55–62. 10.1007/s11060-016-2359-7 28074320

[29] Vandenbroucke JP von Elm E Altman DG Gøtzsche PC Mulrow CD Pocock SJ Strengthening the reporting of observational studies in epidemiology (STROBE). Epidemiology. (2007) 18:805–35. 10.1097/EDE.0b013e3181577511 18049195

[30] Nabors LB Portnow J Ammirati M Baehring J Brem H Butowski N NCCN guidelines insights: central nervous system cancers, version 1.2017. J Natl Compr Cancer Netw. (2017) 15:1331–45. 10.6004/jnccn.2017.0166 29118226

[31] Henegar MM Moran CJ Silbergeld DL Early postoperative magnetic resonance imaging following nonneoplastic cortical resection. J Neurosurg. (1996) 84:174–9. 10.3171/jns.1996.84.2.0174 8592218

[32] Stupp R Brada M van den Bent MJ Tonn J-C Pentheroudakis G High-grade glioma: ESMO Clinical Practice Guidelines for diagnosis, treatment and follow-up. Ann Oncol. (2014) 25(suppl 3):iii93–101. 10.1093/annonc/mdu050 24782454

[33] Sreenivasan S Madhugiri V Sasidharan G Kumar RR Measuring glioma volumes: A comparison of linear measurement based formulae with the manual image segmentation technique. J Cancer Res Ther. (2016) 12:161 10.4103/0973-1482.153999 27072231

[34] Setty P Hammes J Rothämel T Vladimirova V Kramm CM Pietsch T A pyrosequencing-based assay for the rapid detection of IDH1 mutations in clinical samples. J Mol Diagn. (2010) 12:750–6. 10.2353/jmoldx.2010.090237 20847279 PMC2963913

[35] Eads CA Danenberg KD Kawakami K Saltz LB Blake C Shibata D MethyLight: a high-throughput assay to measure DNA methylation. Nucleic Acids Res. (2000) 28:E32 10.1093/nar/28.8.e32 10734209 PMC102836

[36] Sawaya R Hammoud M Schoppa D Hess KR Wu SZ Shi W-M Neurosurgical outcomes in a modern series of 400 craniotomies for treatment of parenchymal tumors. Neurosurgery. (1998) 42:1044–55. 10.1097/00006123-199805000-00054 9588549

[37] Mor V Laliberte L Morris JN Wiemann M The Karnofsky performance status scale. An examination of its reliability and validity in a research setting. Cancer. (1984) 53:2002–7. 10.1002/1097-0142(19840501)53:9<2002::AID-CNCR2820530933>3.0.CO;2-W 6704925

[38] Sanai N Polley M-Y McDermott MW Parsa AT Berger MS An extent of resection threshold for newly diagnosed glioblastomas. J Neurosurg. (2011) 115:3–8. 10.3171/2011.2.JNS10998 21417701

[39] Keles GE Anderson B Berger MS The effect of extent of resection on time to tumor progression and survival in patients with glioblastoma multiforme of the cerebral hemisphere. Surg Neurol. (1999) 52:371–9. 10.1016/S0090-3019(99)00103-2 10555843

[40] Li YM Suki D Hess K Sawaya R The influence of maximum safe resection of glioblastoma on survival in 1229 patients: can we do better than gross-total resection? J Neurosurg. (2016) 124:977–88. 10.3171/2015.5.JNS142087 26495941

[41] Porz N Bauer S Pica A Schucht P Beck J Verma RK Multi-modal glioblastoma segmentation: man versus machine. Strack S, editor. PLoS One. (2014) 9:e96873 10.1371/journal.pone.0096873 24804720 PMC4013039

[42] Chang K Beers AL Bai HX Brown JM Ly KI Li X Automatic assessment of glioma burden: a deep learning algorithm for fully automated volumetric and bidimensional measurement. Neuro Oncol. (2019) 21:1412–22. 10.1093/neuonc/noz106 31190077 PMC6827825

[43] Gahrmann R van den Bent M van der Holt B Vernhout RM Taal W Vos M Comparison of 2D (RANO) and volumetric methods for assessment of recurrent glioblastoma treated with bevacizumab-a report from the BELOB trial. Neuro Oncol. (2017) 19:853–61. 10.1093/neuonc/now311 28204639 PMC5464446

[44] Eidel O Burth S Neumann J-O Kieslich PJ Sahm F Jungk C Tumor infiltration in enhancing and non-enhancing parts of glioblastoma: a correlation with histopathology. Kleinschnitz C, editor. PLoS One. (2017) 12:e0169292 10.1371/journal.pone.0169292 28103256 PMC5245878

[45] Tamura R Ohara K Sasaki H Morimoto Y Yoshida K Toda M Histopathological vascular investigation of the peritumoral brain zone of glioblastomas. J Neurooncol. (2018) 136:233–41. 10.1007/s11060-017-2648-9 29188530

[46] Zinn PO Mahajan B Majadan B Sathyan P Singh SK Majumder S Radiogenomic mapping of edema/cellular invasion MRI-phenotypes in glioblastoma multiforme. Deutsch E, editor. PLoS One. (2011) 6:e25451 10.1371/journal.pone.0025451 21998659 PMC3187774

[47] Ramnarayan R Dodd S Das K Heidecke V Rainov NG Overall survival in patients with malignant glioma may be significantly longer with tumors located in deep grey matter. J Neurol Sci. (2007) 260:49–56. 10.1016/j.jns.2007.04.003 17475281

[48] Henker C Hiepel MC Kriesen T Scherer M Glass Ä Herold-Mende C Volumetric assessment of glioblastoma and its predictive value for survival. Acta Neurochir (Wien). (2019) 161:1723–32. 10.1007/s00701-019-03966-6 31254065

[49] Gutman DA Cooper LAD Hwang SN Holder CA Gao J Aurora TD MR Imaging predictors of molecular profile and survival: multi-institutional study of the TCGA glioblastoma data set. Radiology. (2013) 267:560–9. 10.1148/radiol.13120118 23392431 PMC3632807

[50] Chahal M Xu Y Lesniak D Graham K Famulski K Christensen JG MGMT modulates glioblastoma angiogenesis and response to the tyrosine kinase inhibitor sunitinib. Neuro Oncol. (2010) 12:822–33. 10.1093/neuonc/noq017 20179017 PMC2940678

[51] Molinaro AM Hervey-Jumper S Morshed RA Young J Han SJ Chunduru P Association of maximal extent of resection of contrast-enhanced and non-contrast-enhanced tumor with survival within molecular subgroups of patients with newly diagnosed glioblastoma. JAMA Oncol. (2020) 6:495–503. 10.1001/jamaoncol.2019.6143 32027343 PMC7042822

[52] Boxerman JL Zhang Z Safriel Y Rogg JM Wolf RL Mohan S Prognostic value of contrast enhancement and FLAIR for survival in newly diagnosed glioblastoma treated with and without bevacizumab: results from ACRIN 6686. Neuro Oncol. (2018) 20:1400–10. 10.1093/neuonc/noy049 29590461 PMC6120359

[53] Egger J Kapur T Fedorov A Pieper S Miller J V, Veeraraghavan H, et al. GBM volumetry using the 3D slicer medical image computing platform. Sci Rep. (2013) 3:1364 10.1038/srep01364 23455483 PMC3586703

